# Development of an In Vivo Sensor to Monitor the Effects of Vapour Pressure Deficit (VPD) Changes to Improve Water Productivity in Agriculture

**DOI:** 10.3390/s19214667

**Published:** 2019-10-28

**Authors:** Filippo Vurro, Michela Janni, Nicola Coppedè, Francesco Gentile, Riccardo Manfredi, Manuele Bettelli, Andrea Zappettini

**Affiliations:** 1 Istituto dei materiali per l’elettronica e il magnetismo (IMEM-CNR) Parco Area delle Scienze 37/A, 43124 Parma, Italy; filippo.vurro@imem.cnr.it (F.V.); nicola.coppede@imem.cnr.it (N.C.); riccardo.manfredi@imem.cnr.it (R.M.); manuele.bettelli@imem.cnr.it (M.B.); andrea.zappettini@imem.cnr.it (A.Z.); 2 Istituto di Bioscienze e Biorisorse (IBBR-CNR) Via Amendola 165/A, 70126 Bari, Italy; 3 Department of Electrical Engineering and Information Technology, University Federico II, 80138 Naples, Italy; francesco.gentile2@unina.it

**Keywords:** OECT, biosensors, VPD, water scarcity, tomato

## Abstract

Environment, biodiversity and ecosystem services are essential to ensure food security and nutrition. Managing natural resources and mainstreaming biodiversity across agriculture sectors are keys towards a sustainable agriculture focused on resource efficiency. Vapour Pressure Deficit (VPD) is considered the main driving force of water movements in the plant vascular system, however the tools available to monitor this parameter are usually based on environmental monitoring. The driving motif of this paper is the development of an in-vivo sensor to monitor the effects of VPD changes in the plant. We have used an in vivo sensor, termed “bioristor”, to continuously monitor the changes occurring in the sap ion’s status when plants experience different VPD conditions and we observed a specific R (sensor response) trend in response to VPD. The possibility to directly monitor the physiological changes occurring in the plant in different VPD conditions, can be used to increase efficiency of the water management in controlled conditions thus achieving a more sustainable use of natural resources.

## 1. Introduction

Global food demand is increasing as the world population expands to some 10 billion people by 2050. Land and water resources and the way they are used are central to the challenge of improving food security across the world and to address the impact of ongoing climate change [1]. A major drawback of climate change is water scarcity in which farming plays a major role since agriculture accounts for 70% of the fresh water withdrawals, with an estimated increase to 2.9 thousand km^3^ by 2050 [2].

Tomato (*Solanum lycopersicum* L.) is one of the most important crops worldwide, with a production of approximately 182 × 10^6^ metric tons and a harvested area of more than 4 × 10^6^ ha [3]. Notwithstanding the adaptability of tomato to a wide variety of climates, horticultural production is concentrated in a few warm and rather dry areas: about 34% of world production comes from countries around the Mediterranean sea [4] where Italy is the 7th largest producer, accounting for 59% and 14% of the European and total world production, respectively. Tomato plants need a considerable supply of water throughout the growing period for optimal quality and higher yield, and to prevent yield losses in case of drought occurring during flowering and fruit development [5]. Thus, trends are shifting from emphasizing production per unit area towards maximizing the production per unit of water consumed [6], hence the focus on water use efficiency. However, land and water availability are strong determinants for agriculture, and it is estimated that 50% of fresh vegetables are grown in protected conditions [7]. The greenhouse environment, if properly managed, can significantly increase yield and quality; in fact, more than 1560 world producers choose controlled environment for vegetables cultivation, with 405,000 hectares of greenhouse space dedicated to vegetable production worldwide [8]. 

In the upcoming years the application of greenhouse automation, sensors, and distributed and pervasive computing will allow provision of the optimal growth and cultivation conditions for vegetables [9]. The technological level of greenhouse cultivation, especially in the Mediterranean countries, is low. 

Amongst the parameters that can be controlled, VPD plays a major role in estimating the real loss of water by the plant and for increasing plant water use efficiency notwithstanding it’s indirect evaluation through the measure of air temperature and relative humidity [10]. Water use efficiency, refers not only to the photosynthetic activity of the plant but also to its capacity to manage the amount of water that is available in the soil in order to sustain plant transpiration, particularly under water limited environments [11]. From a physics perspective, water transport along the soil-plant-atmosphere continuum is a passive process driven by gradients of free energy. The driving force for water movement is the transpiration rate that is determined by changes in VPD along the gas phase (from internal leaf to the atmosphere) and is expressed as a combined function of air temperature and relative humidity [12,13,14,15]. Previous papers investigated the effects of VPD on the transpiration rate [16] and on plant growth (mainly on decreased leaf area) [17,18] and highlighted that VPD regulation can improve water use efficiency, with concomitant improvements in biomass and fruit production [19]. The possibility to monitor the effects of VPD changes is becoming of great interest in greenhouses with technology developers aiming to provide fine regulation of atmospheric moisture to positively affect the reduction of water consumption and improve water use efficiency under cultivation.

However, so far, few remote and proximal devices have been tested and are available to monitor the environmental conditions as humidity, temperature, lux, and CO_2_ content (see Table 1 for an overview) and in turn to assess the VPD. The implementation of tools to correlate the effects of VPD environmental on the plant physiology is mandatory to increase the efficiency of indoor growth and production. This is why technical developers and controlled conditions platform operators are demanding low cost sensors able to monitor in real plant/environment conditions.

In the sensors scene, organic electrochemical transistors (OECTs) are promising devices for in vitro and in vivo plant physiology trait measurement applications [20,21,22,23,24,25,26,27]. An OECT is a three-terminal device in which a conducting polymer channel is deposited on the source and the drain, while the gate is separated from the channel by an electrolyte [26].

Recently, an in vivo sensor based on an organic electrochemical transistor (OECT), termed as “bioristor”, has been developed on textile thread and demonstrated to be able to monitor, directly within the plant, the changes occurring in the plant sap ionic content under optimal growth conditions but also under drought stress conditions [28,29,30]. Research activities using OECTs and their application in plant biology are very active [20,21,22,23,24,25,26,27].

The focus of this paper is to gain additional information on how VPD can affect the ion status in the plant sap and to establish a correspondence between the R (the response of the OECT) and a physical characteristic of the system, paving the way for the use of bioristors as an innovative tool to achieve a sustainable use of natural resources.

## 2. Materials and Methods

### 2.1. Plant Material and Growth Conditions

Five tomato plants (*Solanum lycopersicum* L.) cultivar Ikram were grown in 2.6 L pots up to the initial phase of flowering development [19] 0.4 m^3^ h cabinet, under a 16 h photoperiod; the Relative Humidity (RH) ranged from 55–70%. The growth chamber was equipped with a EasyLog datalogger (Lascar Electronics Ltd., Salisbury, UK) to monitor and register constantly the temperature (T) and RH (Supplementary Table S1).

**Figure 1:**
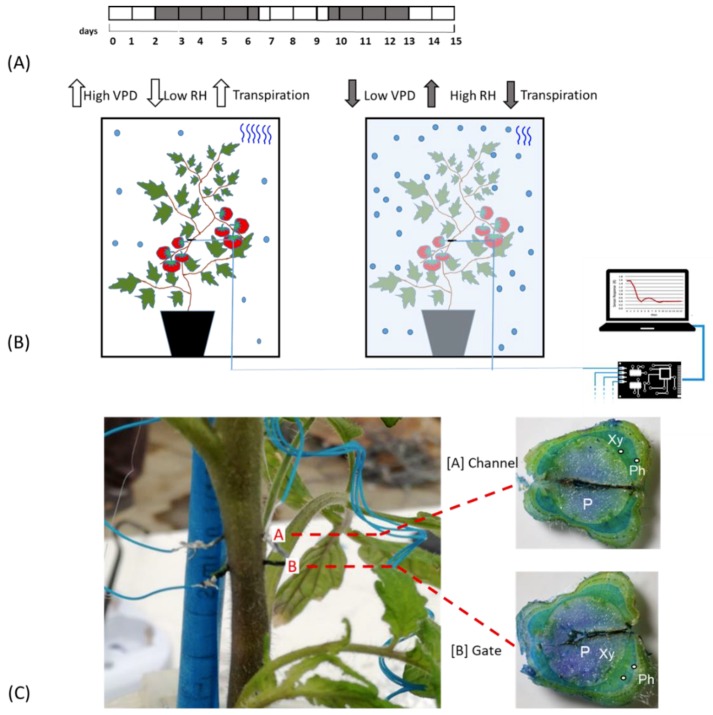
Experimental Outline. (**A**) Time line of the experiment. White blocks indicate the days in which plants are under normal VPD (0.1 and 0.8 kPa); grey blocks indicate the days of low VPD value (between 0.0 and −0.1.; (**B**) Scheme of the bioristor measurements in high (left) and low (right) VPD conditions, the increases or decreases in the expected transpiration rate is also indicated as blue wave in the box; (**C**) Bioristor insertion in a tomato plant: A, channel and, B, Gate. The sections of the stem indicating the tissues crossed by the bioristor are reported and the vascular tissue interested by the bioristor are indicated P, pith; Xy, xylem; Ph, phloem.

Plants were kept fully irrigated until their last phase of vegetative development, after which a bioristor was inserted in the stem of each plant between the third and fourth leaves (Figure 1A,C). 

All plants were irrigated over 2 days post insertion (dpi), and then exposed to low VPD by nebulization of 100 mL of water in the cabinet previously sealed with PVC film (2–7 dpi, Figure 1A). 200 mL of water was supplied to the plants when the growth chamber was opened and plants exposed to an increased VPD (7–10 dpi). From day 10–13 the VPD was again altered to confirm the previously observed mechanisms from13 to15 dpi, Figure 1A).

### 2.2. Physiological Parameter Evaluated

On the basis of the parameters recorded with the datalogger, the *VPD* value was calculated as follows [33,34]:

(1) $$VPD=\left(1-\frac{RH}{100}\right)SVP$$

where *RH* is relative humidity and *SVP* is Saturated Vapour Pressure.

The Saturated Vapour Pressure (SVP) was calculated applying the following equation [33]:

(2) $$SVP=610.7\times 10{\;}^{\frac{7.5T}{237.3+T}}$$

where *T* is the temperature measured in the growth chamber.

### 2.3. Bioristor Preparation and Measurements

The bioristor sensors were prepared as follows. Commercial textile threads were functionalized by soaking them for 5 min in aqueous poly(3,4-ethylenedioxythiophene) doped with polystyrene sulfonate (CleviosPH500, Starck GmbH, Munich, Germany), after which ethylene glycol (10% *v*/*v*) and dodecyl benzene sulfonic acid (12% *v*/*v*) were added. The threads were then baked at 150 °C for 3 h. The final dimensions of treated threads were 1.42 mm × 0.25 mm. A treated thread was inserted through the stem of a tomato plant, with a direct insertion and was cut to a length so that the ends protruded from opposite sides of the stem. The transistor device was completed by introducing the same treated thread acting as a gate electrode (Figure 1C). The electrodes were connected to a NI USB-6343 multifunction I/O device (National Instruments, Austin, TX, USA) [28].

The sensors were inserted into the plant stems between the third and the fourth leaf (Figure 1B) and connected to a computer, following Coppedè et al. (2017) [28]. A constant voltage (V_ds_) was applied across the main transistor channel along with a positive voltage at the gate (V_g_); the resulting currents I_ds_ and I_gs_, representing the current along the main channel and the current flowing through the liquid from the gate to the main channel, were monitored continuously for 15 days. The sensor response R and two time constants, τ and τ_gs_ of the sensor were evaluated (Figure 1B). 

The sensor measures a current of ions, conveyed from the plant to the electrodes. The output of the sensor is a function of time, that smoothly transitions from an initial value ($I_{0}$) to a steady state value ($I_{f}$), similar in shape to the response of a first order system. $I_{0}$ is current measured by the device when $V_{g}=0$. *R* is the increased intensity of the signal compared to its initial value, $R=\left(I_{f}-I_{0}\right)/I_{0}$. The time constant τ, instead, is the time necessary to the sensor to reach the 65% of its final value, and is found upon fitting or the original signal with an exponential function. R is a measure of the strength of the signal and τ is a measure of the rapidity of the signal. Both R and τ are indicative of the characteristics of the system. 

τ and τ_gs_, calculated by fitting the non-linear drain and gate current curves, are related to the time that ions take to enter the polymer, τ, and to the diffusivity of ions in the solution, τ_gs_, respectively [28]. 

Here we considered also the difference I_gs_ = I_gs_ − I_gs0_, where I_gs0_ represented the current across the solution when V_g_ = 0. R, τ, and VPD were mediated over the day to smooth out day/night oscillations. For R and τ parameters the first derivative as a function of time was also calculated to highlight specific trends and indicated as dR and dτ.

### 2.4. Bioristor Biocompatibility

Three plants with the integrated sensor and one control plant were analyzed at the end of the experiment for biocompatibility following the protocol described in Barrs and Weatherley [35]. Sections of stem tissue were prepared using a fresh razor blade and stained with Toluidine Blue O (TBO, Sigma Aldrich, Milano, Italy), a metachromatic stain that produces different colours depending on the polymer to which it adheres. Primary walls (parenchyma, collenchyma, and phloem) are purple and lignified secondary walls of xylem tracheids and vessels (a subtype of vascular tissue) and sclerenchyma are blue, while some other cells may take on a greenish colour. Pictures were acquired with a digital camera equipped with a macro lens.

### 2.5. Statistical Analysis

Data were statistically analysed using R software v3.4.1 package 9 (https://www.r-project.org/). Principal components analysis (PCA) was performed using the “prcomp” function in the R package factoextra (Kassambara and Mundt—R package version, 2016, https://cran.stat.unipd.it/bin/windows/contrib/3.5/) and showed as a biplot. The first two principal components (PC1 and PC2) and the corresponding component loading vectors were visualized and summarized in a biplot, in which component scores (indicated in dots) were coloured according to time classification. PCA was performed for all plants and validated by cluster analyses.

## 3. Results and Discussion

### 3.1. Analyses of the Bioristor Response in Relation to the VPD

The bioristor response (R) was continuously monitored for 15 days in 5 plants in altered Vapour Pressure Deficit (VPD) conditions (Figure 1A,B) and showed a specific trend in sensor response (R) following small changes in the VPD values. Although inserted in the plant stem, he high biocompatibility of the sensor, has been confirmed (Figure 1C). The introduction of bioristor did not alter the overall morphology of the stem and of the plant growth since the plants equipped with the sensors were indistinguishable from other plants not monitored used as control (data not shown). Indeed, the insertion of the sensor did not damage the functionality of the vascular tissues, and even if the vascular tissues were interrupted in the insertion point (Figure 1C), the normal stem structure was restored immediately after the insertion point as also previously reported in Coppedè et al. [28]. Moreover, the use of a textile thread as gate strongly reduced, if compared with the silver one, the onset of necrosis in the tissues surrounding the sensor (Figure 1C).

The day and night trend of R was verified and showed a decrease during the day and an increase during the night tracing the circadian rhythm under normal growth conditions (Supplementary Figure S1) as previously shown in Coppedè et al. [28].

**Figure 2:**
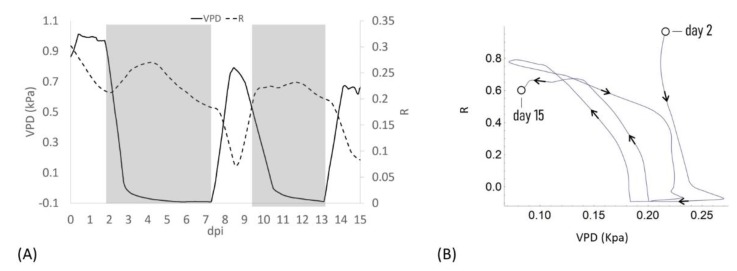
(**A**) Plot of the average of the R measured on all plants for all days post insertion (dpi) at Vg = 1 (dashed line) and the calculated VPD trend (solid line); (**B**) Diagram of the sensor response of the system (R) and the VPD reporting the trajectories described, the arrows indicate the direction of the curve from the beginning to the end of the experiment.

When VPD data were compared with bioristor sensor response, a negative correlation between sensor response R and VPD values was observed (Figure 2). After the expected adaptation period due to sensor integration (days 0–2), when VPD was decreased from 1 to 0.7 Kpa a rapid positive slope of the R was observed for two days (2–4 dpi, 48 h) followed by a smooth decrease of R in constant VPD conditions (Figure 2A).

When VPD was rapidly increased (0–0.7 kPa; day 7.5) R rapidly dropped, showing a complete opposite trend with respect to VPD. When the VPD was decreased (9 dpi) R returned to higher values and remained almost constant, to decrease again when VPD was increased (13 dpi, Figure 2A). When R and the VPD are plotted one as a function of each other and the resulting trajectories are parameterized by time, over approximately 13 days, the curve folds upon itself completing several loops, further indicating that the R and VPD variables are anti-correlated (Figure 2B). 

These data support in vivo the recently reported data on the effects of an alteration of VPD conditions on plant water use efficiency and growth summarized in a strong reduction in the transpiration rate, plant hydraulic conductance, and water flow in general [6]. 

The bioristor data support these findings with the novelty of the acquisition of this information directly at xylem level, continuously and in real time. It is well documented that R increases as a consequence of the increased ionic content of the tested solution, in this case the plant sap [28,29,30].

In low VPD conditions and low transpiration rate, the bioristor always responded with a rapid increase of the R value (2–4 dpi and 9–12 dpi; Figure 2), presumably because of the accumulation of electrolyte (mainly as Na^+^ and K^+^) [36,37] in the xylem sap as consequence of stomatal closure and the subsequent reduction of the transpiration stream [38].

On the contrary, by increasing VPD from 0.1 to 0.8 MPa the transpiration stream seems to be restored with a reduction of the concentration of ions in the xylem that is evidenced by the bioristor response (rapid increase of R). To validate these observations a principal component analysis (PCA) was performed. 

**Figure 3:**
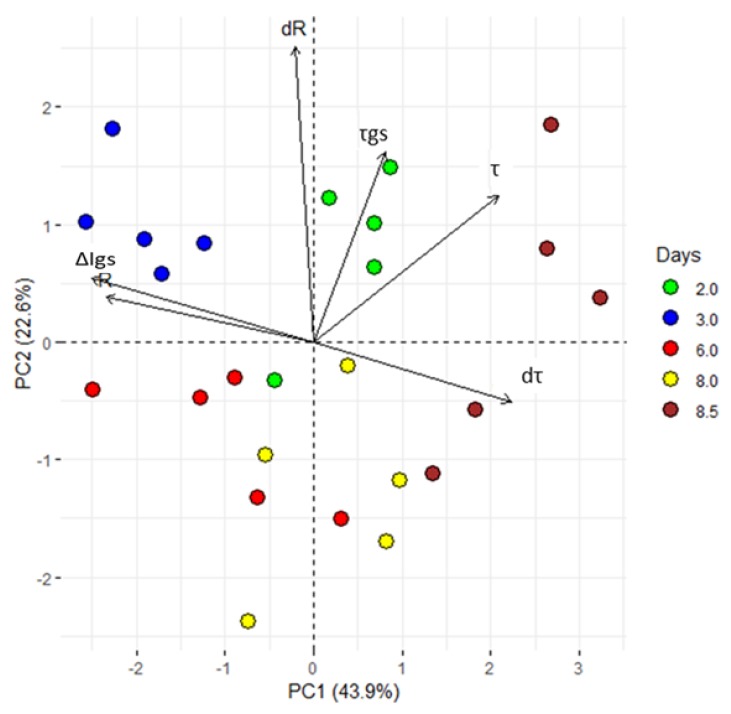
Principal component analysis (PCA). ∆I_gs_; difference between minimum and maximum current gate values, τ and τ_gs_; time constant. From the acquired data, the first derivative of R and τ (dR and dτ) was calculated. All values, exhibited clear separation between the groups with different periods of exposure to VPD variations (2, 3, 6, 8 and 8.5 dpi). The first two components PC1 and PC2 explain the 66.5% of the variability observed. Each dot represents a plant.

PCA is a statistical technique of analysis that reduces the dimensionality of a data-set still retaining much of its informative content. After PCA, a signal is decomposed into a few variables (termed principal components), that are representative of the state of the system. In Figure 3, we report the system’s response expressed as a function of the first two principal components, i.e., PC1 and PC2. Each point in the diagram describes the state of the system measured at specific times. Samples measured at the same time have the same colour. For the analysis, we have considered 5 different time groups (i.e., days 2, 3, 6, 8 and 8.5), and 5 samples (plants) for time group.

PCA shows that the first two principal components explain the 66.2% of the total variance. Moreover, a clear separation of PC variables for all considered times (Figure 3) confirm the hypothesis that alterations in the VPD are correctly tracked by the sensor and encoded in the system’s response, R. After PCA, sample points are clearly separated in clusters: thus, the technique correctly operates sample classification on the basis of their originating time of measurement. Since the time at which a sample is measured encodes information about the VPD history of a plant, the fact that the PCA (that is a mathematical transformation of the signal R discriminates between different time steps automatically implies that the response R is indicative of VPD changes, confirming the initial hypothesis. The groups including 8 and 8.5 dpi can be considered as an individual super-group, where the plants responded to a VPD increase.

The analyses of the correlation between R and VPD at 15 dpi (ρ = −0.80; *p* ≤ 0.05) further supports the anti-correlation between the two variables and the ability of bioristors to sense changes in VPD.

In addition, the plant-to-plant variability in terms of VPD-R-correlation was verified and the single plant R and VPD trends were measured for the entire length of the experiment (Supplementary Figure S2a). No significant difference was observed between all plants considered or in the average of the sensor response over 15 days (about 60 measurements per day). 

**Figure 4:**
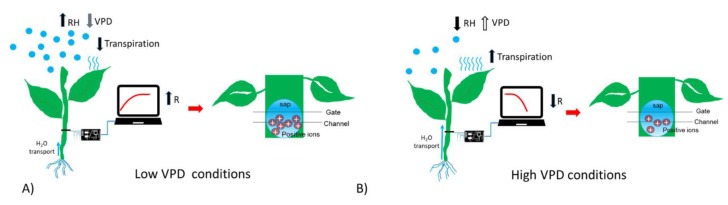
Scheme reporting the sensor response (R) in (**A**) low VPD conditions; (**B**) high VPD conditions.

To summarize, in presence of high humidity and low VPD, thus low transpiration conditions, the bioristor showed a positive increasing trend (high R, Figure 4A); while in low humidity, and high VPD thus high transpiration the bioristor response showed a minimum (Figure 4B).

### 3.2. Analyses of the Bioristor Time Response τ in Relation to the VPD 

The electrolyte (in this case the plant sap) is an integral part of the OECT device; variations in its ionic concentration affect the device properties [30,39]. Another parameter that can be affected by changes in the plant sap ion composition and concentration is τ that gives the time of how fast the channel of the OECT will be de-doped and is directly linked to the diffusion properties of charged species in electrolyte solution (atomic mass, net charge, diffusion coefficient). τ was also acquired to give further information on the ionic composition of the plant sap [28,29,40].

**Figure 5:**
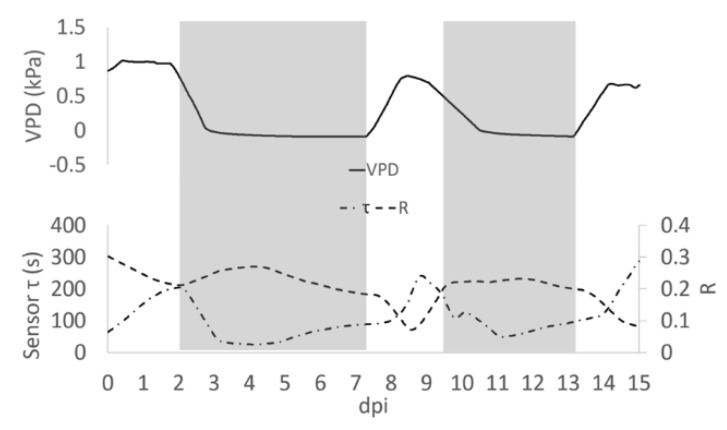
Average of R measured during the day post insertion (dpi) on all plants at Vg = 1 (dashed line), τ signal (dashed and pointed line) and the calculated VPD trend (solid line). Grey block indicates when the Low VPD conditions were applied.

**Figure 6:**
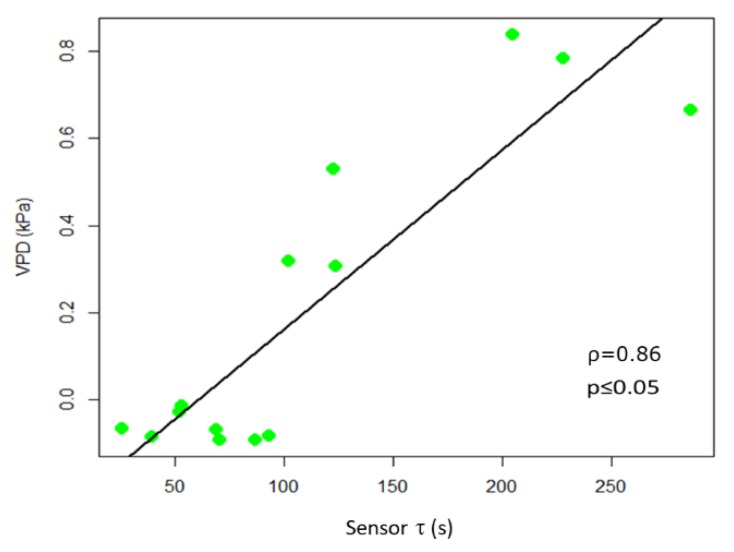
Scatter plot of τ and VPD. The scatter plot and linear regression indicate a high positive correlation between the two variables, with Pearson correlation coefficients ρ = 0.86, *p* ≤ 0.05.

In observing the trend of τ in relationship to R, we noted (Figure 5) that in some portions of the diagram they are correlated, being in an inverse relationship, while in other portions there is a poor correlation between variables. Recalling that R is related to the quantity of ions in solution, while τ is related to the inverse of both ion quantity and mass [26], we can observe an increasing R value is indicative of solutions becoming enriched with more ions of the same type. Moreover, we reported an increasing value of τ, without a correlation with a decrease of R that is indicative of solutions becoming enriched with ions with larger mass. The slope of R and τ determined as a function of time, indicates whether the transformation of the system is of the first (1) or second (2) type, or a combination of the two τ and the VPD trends were comparable (Figure 5). A consistent positive correlation between τ and the VPD values was observed (ρ = 0.86; *p* ≤ 0.05; Figure 6).

**Figure 7:**
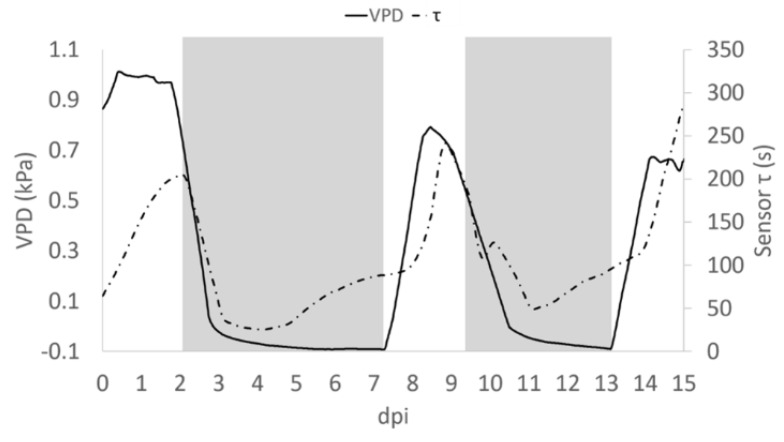
Average trend of τ (dashed pointed line) and VPD (solid line). Grey block indicates when the Low VPD conditions were applied.

A close analysis of the time dependence of τ and VPD, allows the shift of τ and, with the VPD phase in advance by ~12 h (Figure 7). This may be attributed to the different diffusivity of the ions dissolved in the solution, suggesting that bioristor reveals the changes occurring in the ion uptake, storage and distribution triggered by the plant in altered environmental conditions. To support this 12 h time lag, we measured the degree of similarity of the VPD and τ functions using cross correlation. We applied a varying displacement ϕ between the VPD and τ functions, then we calculated the cross correlation between functions as their inner product. The resulting cross correlation is displayed in Supplementary Figure S3 as a function of ϕ. The value of ϕ in correspondence of which the cross correlation is peaked, indicates the lag between VPD and τ. For this configuration, the lag is of nearly 12 h.

**Figure 8:**
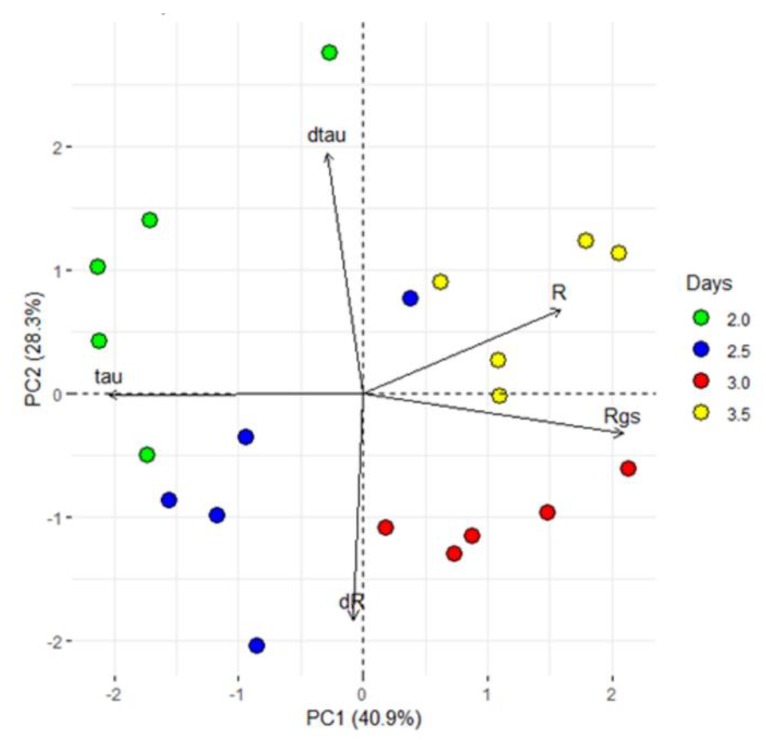
Principal component analysis (PCA). PCA used the processed data, which consist of the sensor response (R), the difference between minimum and maximum current gate values (∆I_gs_), the sensor constants time (τ and τgs) and the first derivative as function of time of R and τ (dR and dτ) and exhibited clear separation between the groups with different periods of exposure to VPD variations (2, 2.5, 3 and 3.5 dpi). The first two components PC1 and PC2 explain the 69.2% of the variability observed.

In view of the reported sensor features (R and τ), a closer analysis of the first 84 h (3.5 days), characterized by a VPD decrease was done performing a PCA analyses. The first two PCs explain the 69% of the total variance and the PCA scores are separated into four groups. In particular, the loading directions indicated that the first two groups were more influenced by τ (thus on the type of ion dissolved in the sap) than the others (Figure 8).

## 4. Conclusions

This paper presents the bioristor as a novel tool for real time monitoring of the changes occurring in the plant sap following changes in VPD conditions. We have shown how a bioristor can dynamically monitor the physiological changes correlated with the regulation of the VPD and that the sensor response showed an opposite trend with VPD. The results achieved in this research paper supports in vivo the hypothesis that the reduction of the leaf transpiration rate in low VPD conditions leads to the accumulation of ions in the early phases of the plant response, followed by a decrease in ionic concentration due to re-opening of stomata under high VPD conditions [6,37,41,42]. During reversible transitions in VPD conditions we identified a clear and unique trend of the R signal, giving a direct and immediate response of the sensor from the inside of the plant system. The use of the bioristor as a smart sensor in greenhouse conditions to fine tune the regulation of VPD can be used to achieve increased water use efficiency and yield.

## Figures and Tables

**Table 1:** Climate remote sensors for environment measuring of VPD.

Sensors	Type of Sensor	References or Web Link	Technical Notes
Smart Bee system	Remote	https://hightimes.com/grow/understanding-vapor-pressure-deficit/	Measure of air temperature and humidity
Microcontroller run in Arduino	Remote	Ramos-Fernandez et al., 2016 [31]	Fuzzy modelling
Pointed Microclimate sensor	Proximal	https://www.30mhz.com/industry/agriculture/	Infrared temperature sensor + vented temp/humidity sensor
Smart sensor	Remote	Millan-Almaraz et al., 2010 [32]	Air temperature, leaf temperature, air relative humidity, plant out relative humidity and ambient light
Pulse One	Remote	https://getpulse.co/	Remote monitoring of temperature, RH, light, and VPD
Micro Grow’s Water Pro	Remote and proximal	https://microgrow.com/	Irrigation controller through environmental monitoring with 11 sensors. VPD is included and estimated by temperature and relative humidity
Digital infrared thermometer (Model GM320)	Proximal	Zhang et al., 2017 [6]	Measure of the leaf temperature
ATMOS 14	Climate remote sensors	www.growlink.com	Temperature, relative humidity, barometric pressure, and vapour pressure

## Supplementary Materials

The following are available online at https://www.mdpi.com/1424-8220/19/21/4667/s1, Figure S1: Day and night modulation of the bioristor response R at different gate voltage (blu: 1V, red 0.6 V, Figure S2: (**a**) Plot showing the average of the individual plant R measured at Vg = 1 (Plant 1-5, dashed coloured lines) and the calculated VPD trend (black line); (**b**) Average and standard deviation of R values for each plant (coloured bars) and considering all plants (dark blu bar) performed over 15 days. (**c**) Scatter plots of the individual plant R and VPD. The scatter plot indicated a high negative correlation between the two variables for plant 1 and 2, and a strong negative correlation for plant 3 and 4 (p ≤ 0.05), the total correlation coefficient ρ is also indicated (dark blue bar). Figure S3 The VPD and time constant (τ) functions measured as a function of time (A). Of the originating functions, we isolated the portion of the functions curves around the maximum (B). We then performed the cross correlation between functions, as a function of the arbitrary time lag ϕ: the peak of the cross correlation indicates the lag between functions that, for this configuration, is approximately of 12 h (C), Figure S4 Transfer characteristics of the R measured using different concentrations of Sodium (Na+), Potassium (K+), Calcium (Ca2+) and Magnesium (Mg2+) salts expressed as the sensor response (R), where I is the off current (measured for gate voltages, Vg ≠ 0 V) and I0 is the on current (measured for Vg = 0 V), Table S1: Daily average of Temperature (T), Relative Humidity (RH) and Vapour Pressure Deficit (VPD) for the 15 days of the experiment. VPD was calculated as reported in Materials and Methods.
