# Supplementary material for: Development of an In Vivo Sensor to Monitor the Effects of Vapour Pressure Deficit (VPD) Changes to Improve Water Productivity in Agriculture
Source: Sensors (Basel). 2019 Oct 28;19(21):4667. doi: 10.3390/s19214667 (PMC6864644; doi:10.3390/s19214667)
Supplement: Supplementary file 1 [file sensors-19-04667-s001.pdf]

sensors

Supplementary file

Development of an in vivo sensor to monitor the effects of Vapour Pressure Deficit (VPD) changes to improve water productivity in agriculture.

Filippo Vurro[1], Michela Janni*[1,2], Nicola Coppedè[1], Francesco Gentile[3], Riccardo Manfredi[1], Manuele Bettelli[1] and Andrea Zappettini[1]

[1]IMEM-CNR Parco Area delle Scienze 37/A-43124 Parma
[2] IBBR-CNR Via Amendola 165/A 70126 Bari
[3]Department of Electrical Engineering and Information Technology, University Federico II, Naples, Italy
* Correspondence: ichela.janni@imem.cnr.it

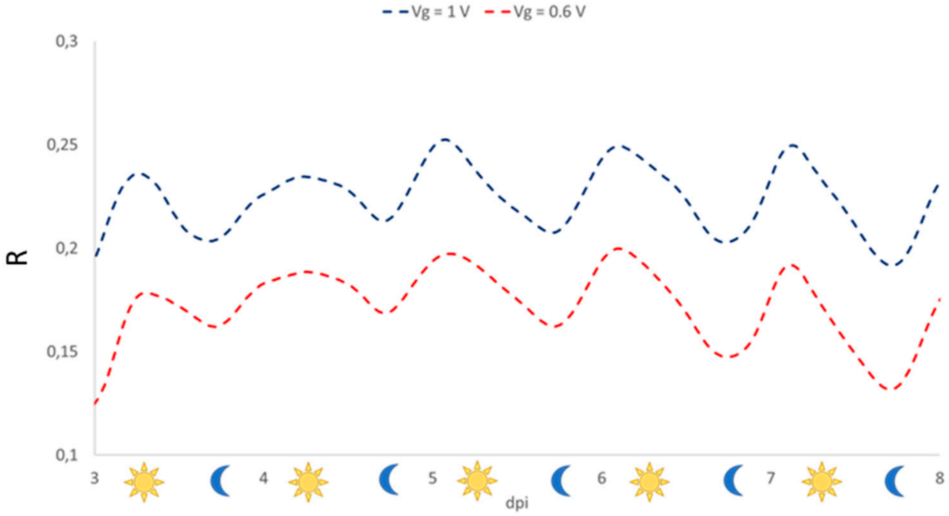

Figure S1. Day and night modulation of the bioristor response R at different gate voltage (blu: 1V, red 0,6 V).

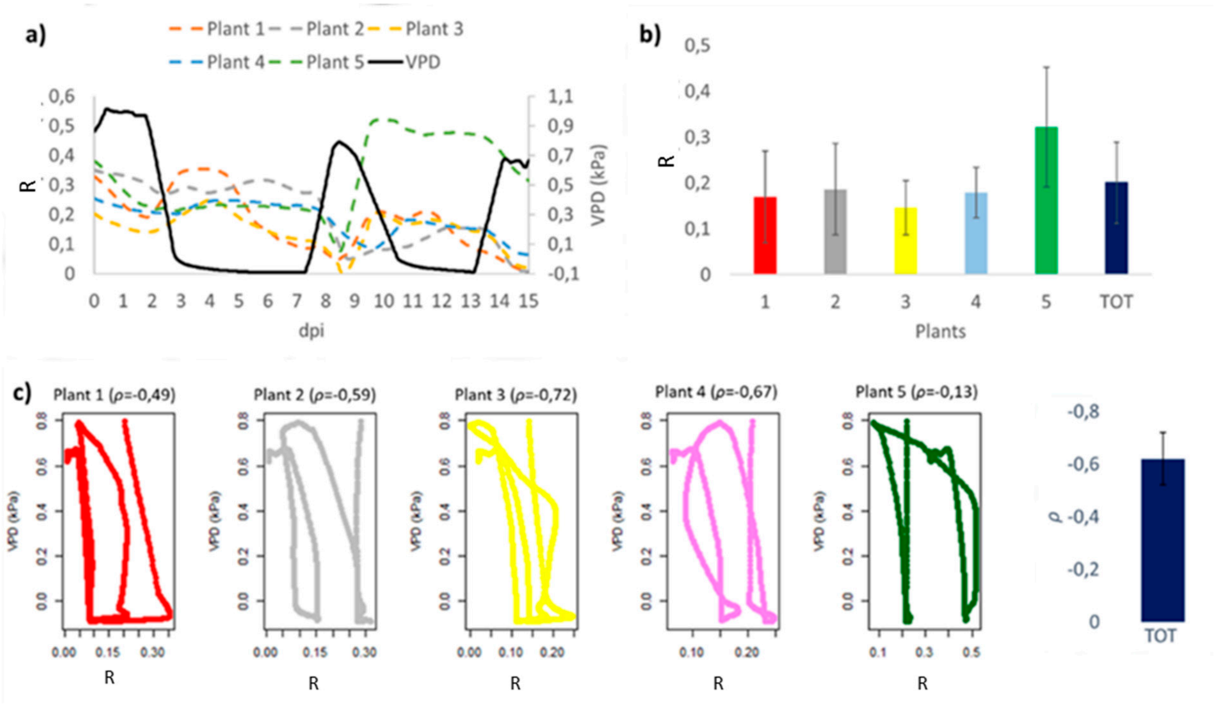

Figure S2: (**a**) Plot showing the average of the individual plant R measured at Vg=1 (Plant 1-5, dashed coloured lines) and the calculated VPD trend (black line); (**b**) Average and standard deviation of R values for each plant (coloured bars) and considering all plants (dark blu bar) performed over 15 days; (**c**) Scatter plots of the individual plant R and VPD. The scatter plot indicated a high negative correlation between the two variables for plant 1 and 2, and a strong negative correlation for plant 3 and 4 ($p \leq 0.05$), the total correlation coefficient ρ is also indicated (dark blue bar).

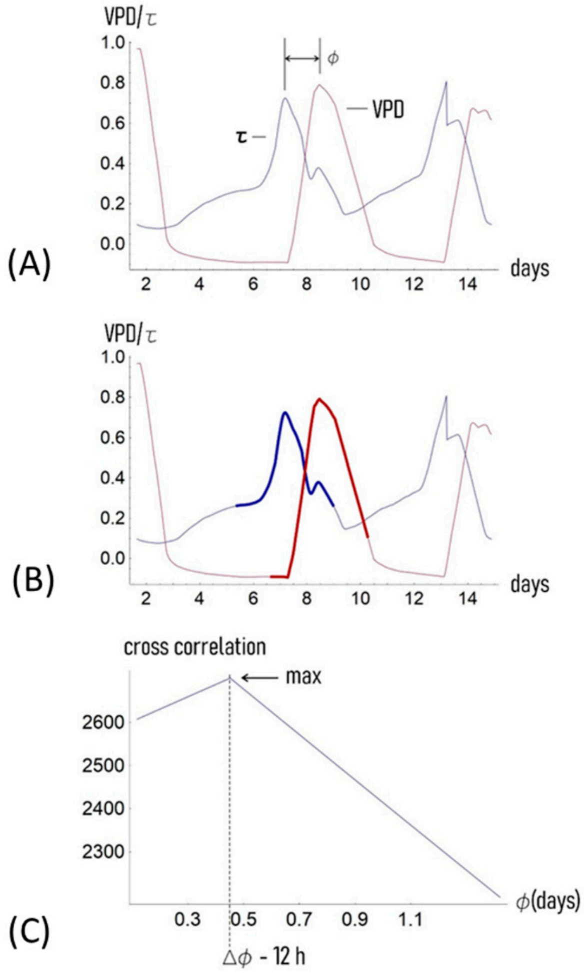

Figure S3. The VPD and time constant (τ) functions measured as a function of time (A). Of the originating functions, we isolated the portion of the functions curves around the maximum (B). We then performed the cross correlation between functions, as a function of the arbitrary time lag ϕ: the peak of the cross correlation indicates the lag between functions that, for this configuration, is approximately of 12 h (C).

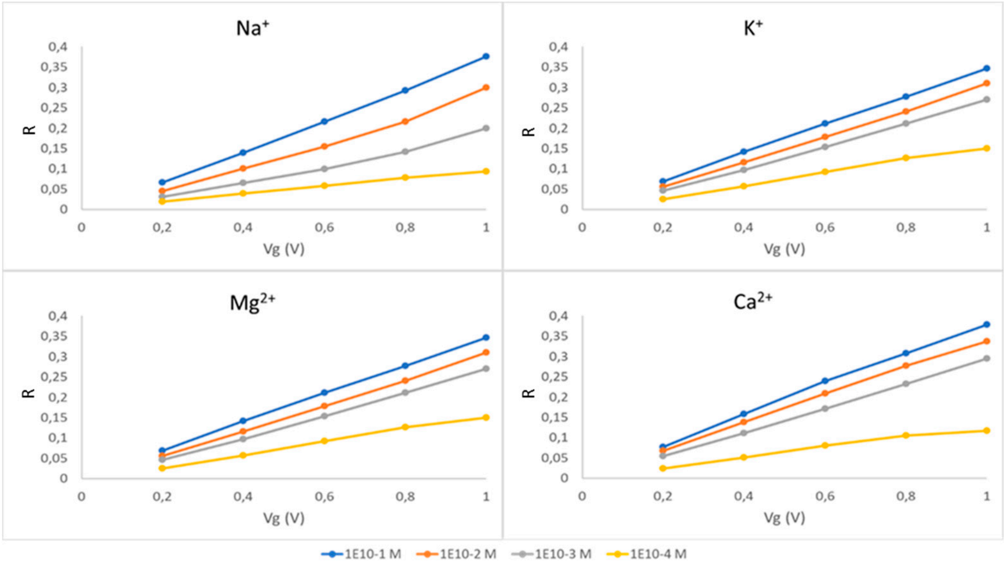

Figure S4 Transfer characteristics of the R measured using different concentrations of Sodium (Na+), Potassium (K+), Calcium (Ca2+) and Magnesium (Mg2+) salts expressed as the sensor response (R), where I is the off current (measured for gate voltages, Vg ≠ 0 V) and I0 is the on current (measured for Vg = 0 V).

Table S1. Daily average of Temperature (T), Relative Humidity (RH) and Vapour Pressure Deficit (VPD) for the 15 days of the experiment. VPD was calculated as reported in Materials and Methods.

Days	T (°C)	RH (%)	VPD (Kpa)
0	23,093	63,376	0,864
1	22,292	55,813	0,993
2	22,875	63,712	0,838
3	24,386	100,566	-0,014
4	23,757	102,733	-0,066
5	23,743	103,451	-0,084
6	23,462	103,816	-0,091
7	22,566	103,972	-0,090
8	21,917	85,128	0,320
9	21,077	62,786	0,784
10	21,449	75,313	0,530
11	22,382	101,208	-0,027
12	22,715	102,917	-0,067
13	23,331	103,503	-0,083
14	23,007	86,625	0,307
15	22,644	71,000	0,664
